# Single Cell Oxygen Mapping (SCOM) by Scanning Electrochemical Microscopy Uncovers Heterogeneous Intracellular Oxygen Consumption

**DOI:** 10.1038/s41598-017-11956-w

**Published:** 2017-09-12

**Authors:** Carla Santana Santos, Alicia J. Kowaltowski, Mauro Bertotti

**Affiliations:** 1 Departamento de Química Fundamental, Instituto de Química, Universidade de São Paulo, São Paulo, 05513-970 Brazil; 2 Departamento de Bioquímica, Instituto de Química, Universidade de São Paulo, São Paulo, 05513-970 Brazil

## Abstract

We developed a highly sensitive oxygen consumption scanning microscopy system using platinized platinum disc microelectrodes. The system is capable of reliably detecting single-cell respiration, responding to classical regulators of mitochondrial oxygen consumption activity as expected. Comparisons with commercial multi-cell oxygen detection systems show that the system has comparable errors (if not smaller), with the advantage of being able to monitor inter and intra-cell heterogeneity in oxygen consumption characteristics. Our results uncover heterogeneous oxygen consumption characteristics between cells and within the same cell´s microenvironments. Single Cell Oxygen Mapping (SCOM) is thus capable of reliably studying mitochondrial oxygen consumption characteristics and heterogeneity at a single-cell level.

## Introduction

Because mitochondrial oxidative phosphorylation is the end-point of most metabolic processes, monitoring oxygen consumption is an effective manner to continuously and non-invasively evaluate energy metabolism in different cell types. Indeed, high-resolution commercial systems have been developed to monitor oxygen consumption in suspended biological samples, using Clark-type electrodes^1–3^, and plated cultured cells^4^, using fluorescent probes. These systems have been successfully used to uncover many different metabolic conditions, with applications as varied as in inherited mitochondrial diseases, inflammation, diabetes, neuroscience and aging^5–9^. Using specific inhibitors, oxygen consumption experiments can determine basal and maximal mitochondrial respiratory capacity, ATP-linked processes, non-ATP-producing respiration (thermogenesis and non-mitochondrial respiration) and estimate substrates used, among other parameters^4, 10^.

However, these techniques present the caveat of detecting only bulk oxygen consumption of the media in which the cells are suspended. They are therefore unable to detect heterogeneity of metabolic characteristics among different individual cells in the same culture, and cannot detect characteristics of this consumption within different areas of a single cell. To date, evaluations of mitochondrial metabolic heterogeneity within and among individual cells have mostly been conducted using fluorescent microscopy and probes for mitochondrial inner membrane potentials. Unfortunately, these evaluations are not quantitative and marred by many artifacts including phototoxicity, influence of plasma membrane potentials, artifacts due to aggregation and changes in mitochondrial mass and morphology^11, 12^.

We thus believe the area would greatly benefit from the development of single cell oxygen consumption techniques. Different techniques have been used to acquire topographical information with high spatial resolution, including atomic force microscopy (AFM), scanning electron microscopy (SEM) and scanning electrochemical microscopy (SECM), which is highly valuable in measurements of local electrochemical activity at interfaces^13–16^. Indeed, SECM has been used in the biological field to uncover enzymatic activities and cellular topography^14, 17–30^. SECM has also been employed to investigate oocyte metabolism and oxygen consumption rates calculated as the difference of oxygen concentrations in the bulk of the solution and close to the cells^31^, estimated according to spherical diffusion theory^31–34^.

In this work, we present an effective and simple approach to evaluate oxygen consumption in the microenvironment of an individual cell, using a platinized platinum disc microelectrode as a tip in SECM configuration. Single Cell Oxygen Mapping (SCOM) experiments were carried out at a fixed tip-cell distance of 15 µm and high spatial resolution information on the oxygen consumption rates was obtained. The results were compared to those acquired with available commercial methods, and show that, while bulk measurements are compatible, our method adds significant spatial distribution information. The use of a platinized platinum microelectrode as a tip in a SECM configuration for mapping the oxygen concentration above a single-cell uncovers rich topographical heterogeneity in oxygen uptake characteristics within individual cells in culture, which may have important regulatory, physiological and pathological implications.

## Results

### Microelectrode development, characterization and calibration

**Figure 1:**
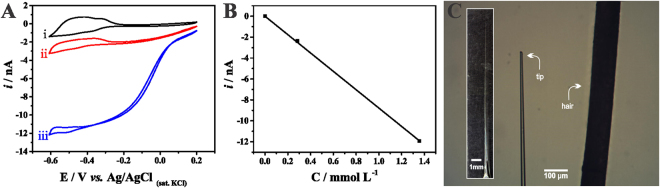
(**A**) Cyclic voltammograms recorded with a platinized Pt microelectrode in argon-saturated (black curve, i), air-saturated (red curve, ii) and O_2_-saturated (blue curve, iii) phosphate buffered saline solution (pH 7.4). Scan rate: 100 mV s^−1^. (**B**) Calibration plot from current values measured at −0.4 V. (**C**) Optical images of the tip compared to a single human hair.

The development of a highly sensitive oxygen microscopy system using SECM included producing platinum disc microelectrodes which were platinized to increase the sensitivity and selectivity of the measurements. The platinization step reduced overpotential for the electrochemical reduction of O_2_ and enhanced the cathodic current, leading to higher sensitivity. Moreover, amperometric responses that were stable over large periods of time were obtained by using the platinized Pt microelectrode. Typical O_2_ electrochemical detection responses of the constructed sensor can be seen in Fig. 1, which shows cyclic voltammograms recorded in the absence of O_2_ (Fig. 1A, black line, i) air-saturated solution (Fig. 1A, red curve, ii) and O_2_-saturated phosphate buffered saline solution (Fig. 1A, blue curve, iii). A steady-state situation is achieved in curves ii and iii, which correspond to the electrochemical process involving O_2_ reduction. Current values at −0.4 V were plotted as a function of O_2_ concentration and the calibration plot is shown in Fig. 1B. The linear plot (R^2^ = 0.99994) shows the constructed platinized Pt microelectrode is a highly sensitive probe to monitor changes in O_2_ concentrations, with a large dynamic concentration range.

### Single Cell Oxygen Mapping (SCOM) by Scanning Electrochemical Microscopy: comparison with commercial systems

**Figure 2:**
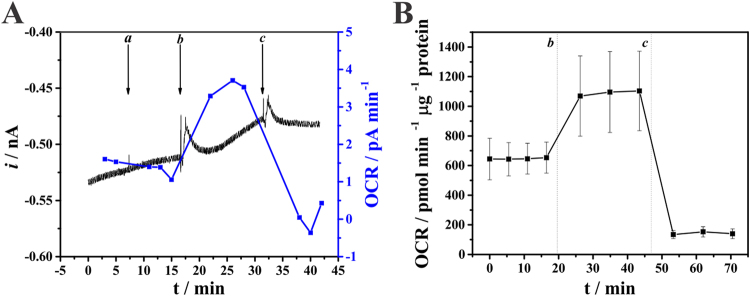
(**A**) Current monitoring using a platinized Pt microelectrode (−0.4 V) positioned at ~15 µm tip-substrate distance from an HS578T cell and O_2_ consumption rate (OCR) values as a function of time. DMEM (2 µL, a), 2 µmol L^−1^ CCCP (b) and 2 µmol L^−1^ antimycin A (c) were added where indicated. (**B**) Oxygen consumption values obtained using a commercial SeaHorse Bioanalyzer under similar experimental conditions.

To investigate cell respiration using the SECM system, the platinized Pt microelectrode was positioned over cultured HS578T cells (chosen for their large surface area) and biased at −0.4 V to monitor changes in local O_2_ concentrations. After 10 minutes for current stabilization, the system displayed a stable current change over time, with good signal to noise relationships. To test for stability upon additions to the system, 2 µL of the culture media DMEM were added (Fig. 2A,a). No alterations in current changes over time were seen, indicating a stable system in which additions could be made. Subsequently, we added known modifiers of cellular oxygen consumption: CCCP (Fig. 2A,b), an uncoupler of oxidative phosphorylation which increases cellular oxygen consumption, and antimycin A (Fig. 2A,c), a specific inhibitor of mitochondrial electron transport and, therefore, oxygen consumption. As expected for these regulators of cellular oxygen consumption, CCCP increased the change in current over time, while antimycin A eliminated it. Control experiments (results not shown) were performed in the absence of cells and no changes were observed upon addition of CCCP and antimycin A, demonstrating that the effects of these drugs were on the cells and not on electrode response. Thus, our SCOM strategy is applicable to cell cultures and presents an adequate response to known regulators of oxidative metabolism.

O_2_ consumption rates were calculated from slope values measured along the recorded black curve shown in Fig. 2A. This representation clearly shows that basal oxygen consumption rates are very significantly increased after the addition of CCCP, whereas these were close to 0 pA min^−1^ in the presence of antimycin A, demonstrating that the SCOM by SECM works well as a respirometer for cultured cells. Indeed, parallel experiments with a SeaHorse respirometer, the gold-standard commercial system available to measure plated cell oxygen consumption, showed similar profiles (Fig. 2B), albeit with more dispersion.

**Figure 3:**
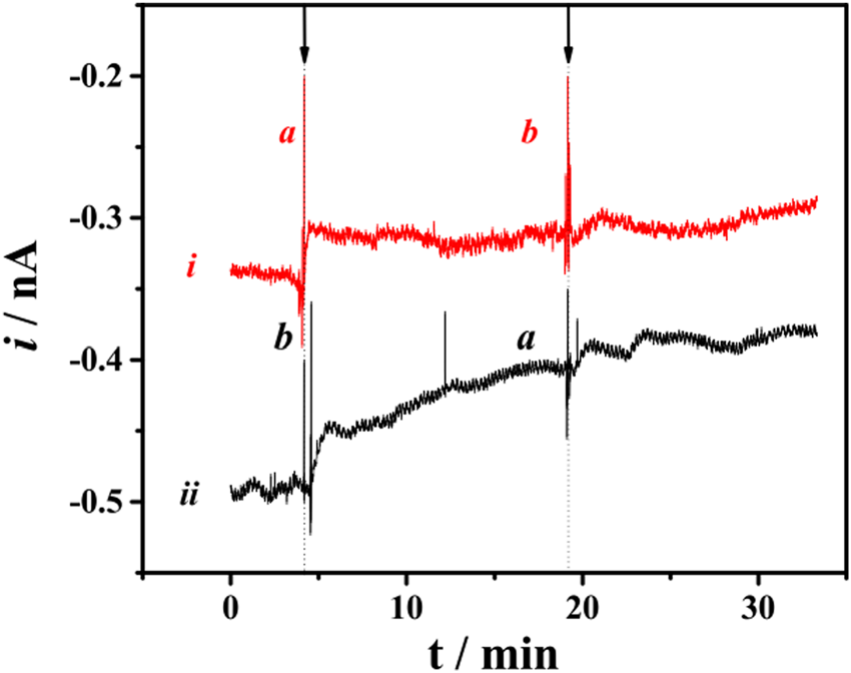
Current monitoring using a platinized Pt microelectrode positioned over HS578T cells. The arrows indicate the addition of antimycin A (a, 2 µmol L^−1^) and CCCP (b, 2 µmol L^−1^). The microelectrode was biased at −0.4 V.

To confirm if the changes in measurements by SCOM were due to the modulation of respiratory activity and not chemical effects of the drugs, the same experiment was repeated, but the substances were added in the opposite order, i.e., antimycin A was added before CCCP. Once complex III is inhibited and the electron transport chain is blocked by antimycin A, the addition of CCCP should not cause any significant change in the respiratory activity. Indeed, the red curve *i* in Fig. 3 shows current changes before and after addition of antimycin A and CCCP, respectively, and no net change in the oxygen consumption rates were observed, confirming the CCCP effect in Fig. 2 was specific to mitochondrial respiration. The experiment was then repeated again under the same conditions in this cell, but CCCP was added before antimycin A; the trace obtained again resembles that in Fig. 2A, with an increase in respiratory activity upon the addition of CCCP. Interestingly, we find that the increase in respiratory activity between individual cells presents some variability (196% and 230%, comparing Figs 2 and 3 curve *ii*). This shows that there are individual characteristics of cellular respiration, which can be uncovered with SCOM, but not commercial systems in which the values obtained are averages for a pool of cells. This is a noteworthy advantage of the proposed approach, since cellular oxygen consumption heterogeneity may be involved in many biological effects, but has not been studied to date.

**Figure 4:**
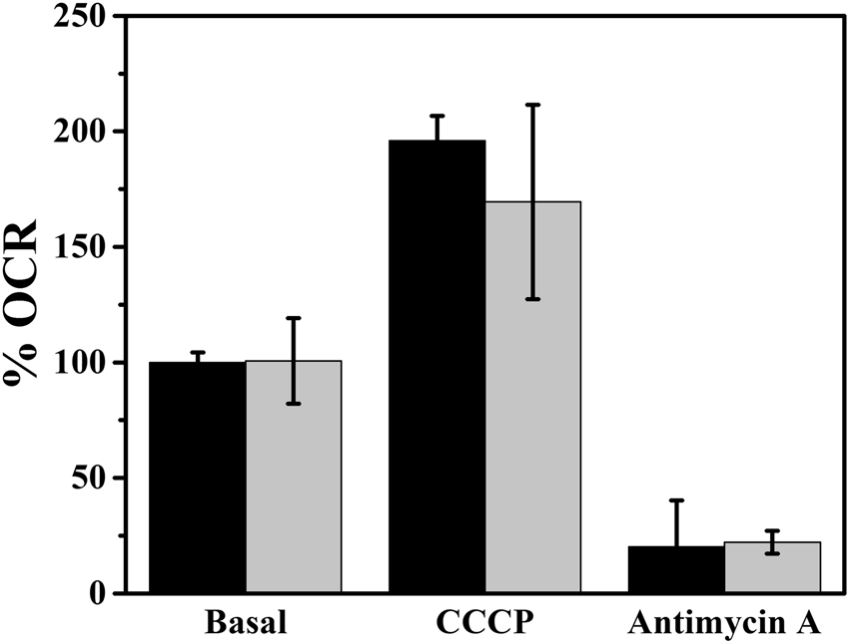
Percentage oxygen consumption rate (OCR) variation for HS578T cells using SCOM (black bars) and SeaHorse (gray bars) respirometers. Basal respiratory rates were used to normalize results, and the effects of CCCP and antimycin A were monitored under conditions described for Fig. 2.

Although the differences in respiratory stimulation seen for specific cells upon the addition of CCCP may be due to individual cell characteristics, current measurements in an SECM system depend on the concentration of the electroactive species as well as the distance between the tip (microelectrode) and the substrate (cell). At the working distance employed here (at around 15 µm using a microelectrode of r = 5 µm), the tip body inhibits lateral oxygen diffusion, acting as a physical barrier (hindered diffusion). Hence, the oxygen concentration in the layer between microelectrode and the cell is controlled by the hindered diffusion effect, the consumption by the tip and the consumption by the cell. Because the rate of electron transfer is the same throughout the experiment (the electrode potential is set at a fixed value) and the working distance is considered constant, these experimental parameters should not cause changes in measured current. Cell height was determined to be 3 µm by atomic force microscopy (AFM), and was thus much smaller than the working distance (15 µm), making any cellular shrinkage or expansion effect negligible. As a result of the hindered diffusion effect, the current decreases as the tip approaches the surface. Thus, the conversion of current into O_2_ concentration is not straightforward, since the accurate distance between the cell and microelectrode surface is difficult to estimate, and could vary in separate measurements. In order to test the reliability of the system, we compared the SCOM/SECM respirometer to the commercial Seahorse respirometer in terms of reproducibility (Fig. 4). Because SCOM measures oxygen consumption in single cells, while Seahorse respirometry measures a cell population, we normalized the readings as percentages of basal oxygen consumption. SeaHorse errors were calculated from measurements performed in four different wells, while the error bars for SCOM measurements correspond to different measurements of a single cell. Interestingly, we noted that variability in the SCOM by SECM was comparable or even lower than the multi-cell Seahorse system (Fig. 4).

### Single Cell Oxygen Mapping (SCOM)

**Figure 5:**
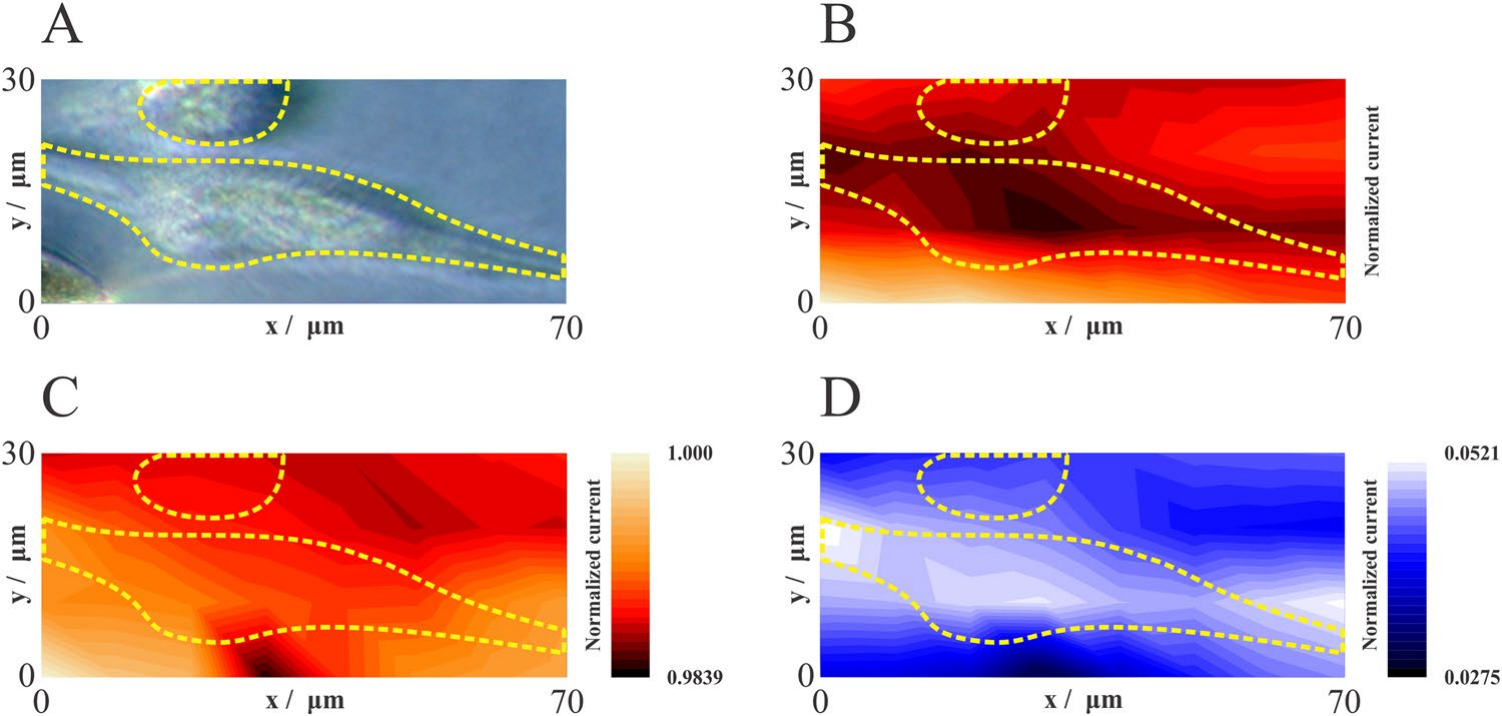
(**A**) Optical image of two cells. Normalized current map of the scanned area before (**B**) and after (**C**) addition of antimycin A (2 µmol L^−1^). For **B** and **C** the highest oxygen reduction current was defined as 1. (**D**) Pixel subtraction map corresponding to normalized current values measured after (map **C**) and before (map **B**) the addition of antimycin A. E = −0.4 V, Pixel size = 10 × 10 µm. The dotted lines show the shape of the cells.

Given the evidence that the SECM system was reliably measuring oxygen consumption, we scanned a submicrometric platinized Pt microelectrode (r = 500 nm) over cultured cells in order to get a map of respiratory activity. In this SCOM image experiment, the microelectrode-substrate distance was set at 15 µm (constant height) and the tip was scanned across the surface of two cells (Fig. 5A), chosen because the lower one presented normal live cell morphology, while the top cell was rounded and seemed to be poorly attached, a characteristic of dead cells, which are not expected to respire. The tip was moved laterally in a horizontal (x-y) plane with 10 µm steps at 5 µm s^−1^. Cyclic voltammograms were recorded only at the end of each step to minimize oxygen depletion^29, 35^. Current values measured at −0.4 V were used to create the SCOM image (Fig. 5). Experiments were performed before (Fig. 5B) and after (Fig. 5C) the addition of antimycin A (2 µmol L^−1^). Current values were normalized (the highest current was defined as 1) and higher upon addition of the inhibitor compared to those values obtained under basal conditions, since oxygen consumption rates decreased.

The result is a map of oxygen consumption in the cell microenvironment, which shows that most of the area of the live cell coincides with areas of oxygen consumption (Fig. 5B). An exception is the area of the nucleus (bulge on the center bottom), which has low consumption, as expected for a cellular area that lacks mitochondria. The dead cell, on the other hand, did not affect oxygen consumption currents. These differences disappear in the antimycin A-treated cell measurement (Fig. 5C), indicating they are caused by oxygen consumption and not topography.

To further compare the inhibitor effect and generate a specific mitochondrial oxygen consumption map, normalized current values measured for intact cells (basal respiration condition) were subtracted from those corresponding to experiments performed in presence of antimycin A (non-mitochondrial respiration condition), generating the pixel subtraction map shown in Fig. 5D. This map compensates for oxygen diffusion effects such as those caused by the mass of the dead cell and indicates areas of mitochondrial oxygen consumption within the live cell (white zones). Our cells are not morphologically similar to spheres and the oxygen consumption is not homogeneous throughout the cell, as shown in Fig. 5B. Hence, spherical diffusion theory^31^ is not appropriate to estimate oxygen consumption rate at our experimental conditions. In an attempt to give the reader information on the quantitative amount of O_2_ molecules consumed in the respiration process as a function of time, we adopted a different approach: O_2_ consumption rates values shown in Fig. 2A were divided by the slope of the calibration plot (obtained at a microelectrode-silicon wafer surface distance of about 15 µm, i.e., a similar microelectrode-cell surface separation), uncovering quantitative changes in O_2_ concentrations at the surface of the cell by unit of time. Taking into account the volume of the solution, the rate of O_2_ consumption was found to be 342 (basal condition), 797 (in the presence of CCCP) and 114 pmol min^−1^ (in the presence of antimycin A). Interestingly, while the cell nucleus area was an expected area of low oxygen consumption, the map (Fig. 5D) shows that cell extremities have very high oxygen consumption, and uncovers heterogeneous oxygen consumption characteristics within a single cell. It should be pointed out that the acquired images have a pixel size resolution of 10 µm, but the scanned area corresponding to the cells extremities is smaller than this value. Even considering this, the extremities of the cell demonstrate significant respiratory activity, leading to a higher oxygen consumption activity zone. In conclusion, the spatial resolution of the SCOM by SECM system thus allows for the visualization of different regions of mitochondrial respiration within a single cell.

## Discussion

Mitochondrial oxidative phosphorylation, as the end-point of energy metabolism, has been extensively studied as a means of understanding metabolic processes underlying physiological and pathological processes. The best way to study oxidative phosphorylation is the measurement of oxygen consumption rates associated with the use of mitochondrial respiratory regulators^1–4^. Oxygen consumption methods are advantageous since they are quantitative, specific and much less artifact-prone than fluorescence microscopy methods to evaluate mitochondrial function^11, 12, 36^. Currently available methodologies to evaluate mitochondrial respiration do so in bulk: tissue samples, isolated mitochondrial homogenates, intact and permeabilized cells^1–4^.

However, recent findings studying mitochondrial parameters other than respiratory rates (such as morphology and inner membrane potentials) suggest there is significant heterogeneity both between different cells in a population and within different mitochondria within the same cell^37–42^. As a result, the development of methodologies capable of measuring oxygen consumption with single cell resolution has become of interest.

Here we developed and validated the use of a SCOM method using SECM and a platinized platinum disc microelectrode with a submicrometric tip (Fig. 1). We have demonstrated the SCOM system is capable of reliably measuring single cell oxygen consumption, responding to mitochondrial respiratory modulators in the expected manner, and displaying stability during measurements and additions (Figs 2 and 3). The system produces results that are comparable, if not better, than the gold-standard currently available commercial system to evaluate respiration in populations of plated cells (Fig. 4), with the advantage of being able to uncover inter-cell variability (Fig. 3). Moreover, the oxygen consumption rate can be assessed in a simpler way than those proposed in the literature^32, 33^, where this parameter was obtained by measuring oxygen concentrations at different distances from the cell surface: This approach has the disadvantage of being influenced by the effect of the working distance on current measurement, since currents also change because of the feedback effect. Finally, when used in a scanning configuration, the SCOM system developed here is able to create oxygen consumption maps, and uncovers heterogeneity in consumption patterns within the same cell (Fig. 5), with enhanced mitochondrial oxygen consumption at the thinner ends of the cell.

We have thus successfully demonstrated the feasibility of non-invasive oxygen consumption microscopy experiments in biological samples, a development that will add to our knowledge of metabolic characteristics on a single-cell basis.

## Methods

### Chemicals

All reagents were of analytical grade and used as received (Sigma Aldrich, USA). Measurements in absence and presence of oxygen were performed in solutions purged with argon and oxygen (Air Products SA – Brazil).

### Microelectrode fabrication and electrochemical characterization

Platinum microelectrodes were fabricated by sealing a 50 µm diameter platinum wire (99.99% purity; hard, Goodfellow, UK) inside a quartz glass capillary (L, 150 mm; o.d., 1.0 mm; i.d., 0.3 mm, Sutter Instruments, USA). The capillary was then pulled using a P-2000 Micropipette Puller (Sutter Instrument Company, USA). An Ag/AgCl (saturated KCl) electrode was used as reference. The microelectrodes were electrochemically characterized by recording cyclic voltammograms and approach curves over a silicon wafer in a 5 mmol L^−1^ [Ru(NH_3_)_6_]Cl_3_ + 0.1 mol L^−1^ KCl solution. The electrochemically-active radius and the RG values (RG = *rg/ r*, where *rg* is the radius of the overall tip, including the active platinum disc and the surrounding insulator; *r* is the radius of the platinum microdisc) of two different microelectrodes were determined by fitting approach curves^43^ before the platinization of the surface. Values were found to be 5 µm (RG = 8) and 500 nm (RG = 15). The smaller microelectrode was used in the SECM image experiment. The calibration plot for oxygen was prepared by using steady-state current values obtained from cyclic voltammograms recorded in a Gibco Dulbecco’s phosphate buffered saline (Thermo Fisher Scientific, USA) solution containing oxygen at different concentrations.

### HS578T cell culture conditions

HS578T human breast cancer cells (ATCC) were grown in plastic flasks in Dulbecco's Modified Eagle's Medium, DMEM, (Thermo Fisher Scientific, USA) supplied with 10% fetal bovine serum, 1% penicillin and streptomycin. Temperature and pH were controlled in an incubator (5% CO_2_ at 37 °C).

### Seahorse bioanalyzer oxygen consumption measurements

HS578T cell oxygen consumption rates were investigated a using a SeaHorse XF 24 Analyzer (Seahorse Biosciences, USA). The experiment was performed in SeaHorse solution containing 5 ^.^ 10^4^ attached cells per well of 24-well Seahorse plates (600 µL). Rates were measured for intact cells (basal respiration conditions) and in the presence of 0.6 µmol L^−1^ carbonyl cyanide m-chlorophenylhydrazoneine, CCCP (maximum respiration) and 2 µmol L^−1^ antimycin A (non-mitochondrial respiration).

### Electrochemical experiments and SCOM

Electrochemical experiments were carried out using an Autolab PGSTAT128 bipotentiostat/galvanostat (Ecochemie, Netherlands) and a Sensolytics (Sensolytics, Germany) SECM coupled to an inverted microscope (Zeiss, Germany). Platinum disc microelectrodes were platinized in a 1 mmol L^−1^ K_2_PtCl_6_ + 0.5 mol L^−1^ H_2_SO_4_ solution by cycling the potential from 0.3 V to −0.5 V vs Ag/AgCl/saturated KCl (100 cycles)^35^. SECM experiments were performed over a Petri dish (TC dish 35, Sarstedt, Germany) filled with 2 mL of DMEM cell culture solution (37 °C, containing 5x10^4^ HS578T plated cells). The tip-substrate distance was determined by performing approach curves at the Petri dish surface close to the cell. The tip was then positioned above the cell. Cell respiration activity was measured with a platinized platinum microelectrode (*r* = 5 µm) polarized at −0.4 V and positioned at a microelectrode-cell surface distance of about 15 µm (hindered diffusion region). Electrochemical images of a single cell were obtained with a platinized platinum microelectrode (*r* = 500 nm) by recording cyclic voltammograms (0.5 V to −0.6 V) after each movement of the tip in the x-y plane. In these experiments, since the SECM tip possessed submicrometer dimensions, it was positioned 15 µm above the cell surface, which is far from the hindered diffusion region. Hence, no topographical effects could be noted at this working distance.
